# Soft Mesoporous Organosilica Nanoplatforms Improve Blood Circulation, Tumor Accumulation/Penetration, and Photodynamic Efficacy

**DOI:** 10.1007/s40820-020-00465-7

**Published:** 2020-06-30

**Authors:** Xin Peng, Kun Chen, Wanhua Liu, Xiongfeng Cao, Mengru Wang, Jun Tao, Ying Tian, Lei Bao, Guangming Lu, Zhaogang Teng

**Affiliations:** 1 Jiangsu Key Laboratory of Molecular and Functional Imaging, Department of Radiology, Zhongda Hospital, School of Medicine, Southeast University, Nanjing, 210009 Jiangsu People’s Republic of China; 2 Department of Medical Imaging, Jinling Hospital, School of Medicine, Nanjing University, Nanjing, 210002 People’s Republic of China; 3 Key Laboratory for Organic Electronics and Information Displays, Jiangsu Key Laboratory for Biosensors, Institute of Advanced Materials, Jiangsu National Synergetic Innovation Centre for Advanced Materials, Nanjing University of Posts and Telecommunications, Nanjing, 210023 People’s Republic of China; 4 Affiliated Hospital of Jiangsu University, Jiangsu University, Zhenjiang, 212001 People’s Republic of China; 5 Soft Matter and Interface Group, School of Engineering, RMIT University, Melbourne, VIC 3000 Australia

**Keywords:** Mesoporous organosilica, Soft nanoplatform, Long circulation, Tumor accumulation, Tumor penetration

## Abstract

To date, the ability of nanoplatforms to achieve excellent therapeutic responses is hindered by short blood circulation and limited tumor accumulation/penetration. Herein, a soft mesoporous organosilica nanoplatform modified with hyaluronic acid and cyanine 5.5 are prepared, denoted SMONs-HA-Cy5.5, and comparative studies between SMONs-HA-Cy5.5 (24.2 MPa) and stiff counterparts (79.2 MPa) are conducted. Results indicate that, apart from exhibiting a twofold increase in tumor cellular uptake, the soft nanoplatforms also display a remarkable pharmacokinetic advantage, resulting in considerably improved tumor accumulation. Moreover, SMONs-HA-Cy5.5 exhibits a significantly higher tumor penetration, achieving 30-μm deeper tissue permeability in multicellular spheroids relative to the stiff counterparts. Results further reveal that the soft nanoplatforms have an easier extravasation from the tumor vessels, diffuse farther in the dense extracellular matrix, and reach deeper tumor tissues compared to the stiff ones. Specifically, the soft nanoplatforms generate a 16-fold improvement (43 vs. 2.72 μm) in diffusion distance in tumor parenchyma. Based on the significantly improved blood circulation and tumor accumulation/penetration, a soft therapeutic nanoplatform is constructed by loading photosensitizer chlorin e6 in SMONs-HA-Cy5.5. The resulting nanoplatform exhibits considerably higher therapeutic efficacy on tumors compared to the stiff ones.

**Electronic supplementary material:**

The online version of this article (10.1007/s40820-020-00465-7) contains supplementary material, which is available to authorized users.

## Highlights

- Soft mesoporous organosilica nanoplatforms modified with hyaluronic acid and cyanine 5.5 are constructed.
- Therapeutic efficacy of the soft nanoplatforms on tumor is higher both in vitro and in vivo after loading photosensitizer chlorin e6 compared to that of stiff counterparts.

**Electronic supplementary material:**

The online version of this article (10.1007/s40820-020-00465-7) contains supplementary material, which is available to authorized users.

## Graphical abstract

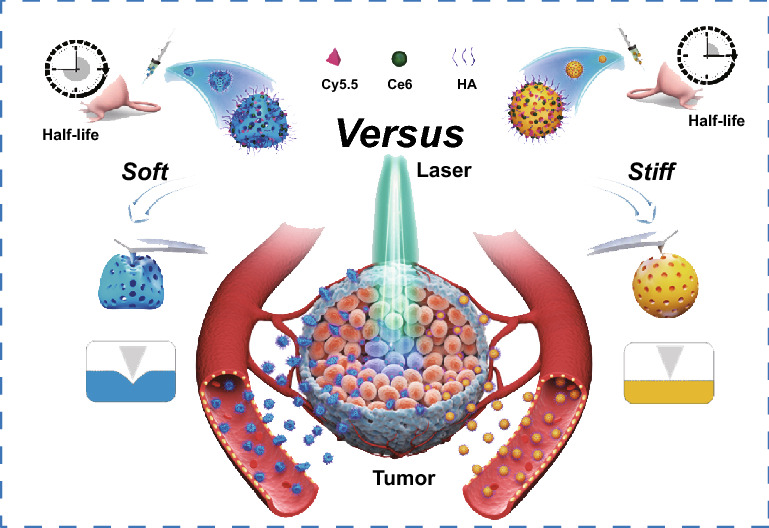

## Introduction

Cancer is a major disease that threatens human life. Recently, nanomedicine has enabled early diagnosis and precise treatment of various tumors, thereby bringing new hope for cancer management [1–4]. Generally, tumors possess an inherent pathologically leaky vasculature and defective lymphatics. Based on these characteristics, nanomaterial-based therapeutic agents that preferentially accumulate in the tumors, via the enhanced permeability and retention (EPR) effect, have been identified and are currently used for cancer treatment, with few side effects [5–8]. However, the nanomaterials systemically administered into the complicated biological systems need to go through multiple barriers, including circulating in the blood compartments, extravasating into perivascular tumor microenvironment and subsequently penetrating into the deep tumor tissues, until they were finally internalized by tumor cells [9–11]. During this complicated process, nanomaterials are likely to be sequestered and eliminated by the mononuclear phagocyte system (MPS) [12], hindered by dense tumor interstitial extracellular matrix (ECM) and the substantially elevated interstitial fluid pressure (IFP) [13, 14]. Consequently, there is a need to rationally design nanoplatforms that can overcome the aforementioned challenges and improve therapeutic outcomes. These platforms are required to meet the following requirements: (1) long blood circulation, (2) high tumor accumulation, (3) deep tumor penetration, (4) efficient internalization in tumor cells. A long blood circulation is one of the key factors for higher tumor accumulation [15, 16]. Meanwhile, deep tumor penetration and efficient cell internalization are essential for improving efficacy of therapeutic nanoplatforms [17–20]. To satisfy the above requirements, numerous platforms have focused on incorporating different physiochemical properties, including shape turning [21–24], size regulation [25–27] and surface modification [28–30] during fabrication of nanomaterials. However, varied approaches all had their own set of limitations, and cannot simultaneously address all issues. To date, short circulation and limited tumor penetration remain bottlenecks for nanoplatforms, thereby preventing effective therapeutic responses.

Mesoporous nanoparticles have become ideal candidates for constructing theranostic nanomaterials, owing to their tunable mesopore structures, sizable surface area and pore volume properties [31–33], that enable them effective vehicles for various molecular imaging probes and anticancer therapeutic agents [34–36]. Recently, research at our lab successfully obtained a soft hollow mesoporous organosilica nanoparticle [37]. Preliminary liquid cell transmission electron microscopy indicated that the nanoparticle undergoes morphological change, from spherical to oval, during cellular internalization, which generates a considerably higher cellular uptake. This advantage inspired us to construct soft nanoplatforms and systemically investigate their biological behaviors including blood circulation, tumor accumulation, tumor tissue penetration and the eventual therapeutic efficacy. To the best of our knowledge, this is the first study comprehensively exploring biological behaviors of soft mesoporous organosilica nanoplatforms.

**Scheme 1:**
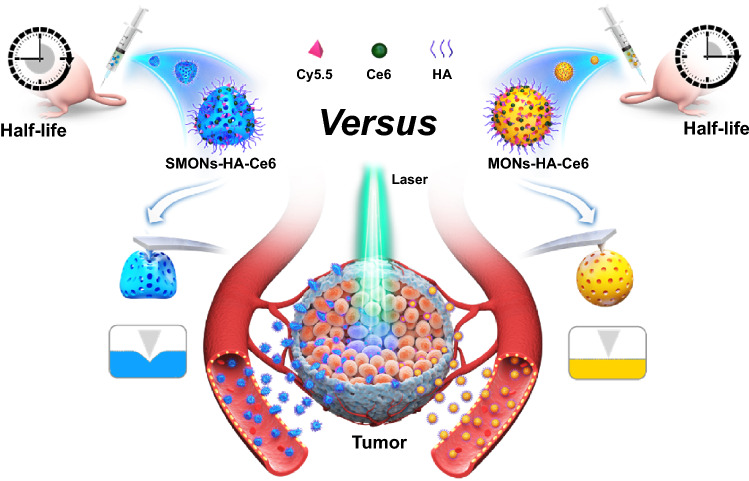
Schematic of the soft nanoparticles as effective anticancer plateforms. The soft nanoplatforms exhibit enhanced tumor accumulation, extravasation from tumor vessels and penetration into deep tumor parenchyma and more efficient antitumor activity due to their softness and deformability compared with their stiff counterparts

In the work, a soft mesoporous organosilica nanoplatform, hereafter termed SMONs-HA-Cy5.5, has been prepared based on hyaluronic acid-(HA) and cyanine 5.5 (Cy5.5)-modification. The outer HA layer comprises nanoplatforms with good dispersibility in aqueous solutions. The soft nanoplatforms have a lower Young’s modulus (24.2 MPa), which is about 31% of the rigid counterparts MONs-HA-Cy5.5 (79.2 MPa). The soft structure grants the SMONs-HA-Cy5.5 double increased cellular uptake in MCF-7 cells and a longer mean residence time (9.09 vs. 3.94 h) in the bloodstream. The significantly elongated blood circulation gives SMONs-HA-Cy5.5 a twofold increase in tumor accumulation compared to MONs-HA-Cy5.5. More interestingly, SMONs-HA-Cy5.5 exhibits 30 μm-deeper tumor penetration, than stiff counterparts in 3D tumor multicellular spheroids in vitro. Analysis of extravasation behavior reveals that SMONs-HA-Cy5.5 have a 16-fold enhancement on diffusion distance in tumor interstitial extracellular matrix, relative to stiff ones that have lower perfusion rates and are thereby restricted in or near the tumor blood vessel. This indicates a significant boost of extravasation of SMONs-HA-Cy5.5 from the tumor blood vessel into the tumor microenvironment. Given the overall improved biological performance of the soft SMONs-HA-Cy5.5, we further load SMONs-HA-Cy5.5 with a photosensitizer chlorin e6, denoted SMONs-HA Ce6. Strikingly, the soft nanoplatform significantly improved the tumor therapeutic efficacy via photodynamic therapy following intravenous injection (Scheme 1). These results suggest that the soft mesoporous nanomaterials have the potential for developing nanotherapeutic platforms with more desirable biological performance and higher therapeutic efficiency.

## Experimental Section

### Materials

Tetraethyl orthosilicate (TEOS), hexadecyltrimethyl ammonium bromide (CTAB, 25 wt%), NaOH, dioxane, and triphenylphosphine were bought from Sinopharm Chemical Reagent Co., Ltd. (Shanghai, China). Bis[3-(triethoxysilyl)propyl] tetrasulfide (TESPTS), Chlorin e6 (Ce6) and *N*-(3-dimethylaminopropyl)-*N*′-ethylcarbodiimide hydrochloride (EDC), and 2′,7′-dichlorofluorescin diacetate (DCFH-DA) were obtained from Sigma-Aldrich (St. Louis, MO, USA). *N*,*N*-dimethylformamide (DMF), concentrated ammonia aqueous solution (NH3H2O, 25–28 wt%) and anhydrous ethanol were obtained from Nanjing Chemical Reagent Co., Ltd. (Nanjing, China). Concentrated hydrochloric acid (HCl, 37 wt%) was brought from Shanghai Jiuyi Chemical Reagent Co., Ltd. (Shanghai, China). Cy5.5-maleimide was obtained from Seebio Biotechnology Co., Ltd. (Shanghai, China). NH_2_-maleimide was purchased from Xi’an ruixi Biological Technology Co. Ltd. (China). *N*-hydroxysulfosuccinimide (NHS) was purchased from Shanghai Aladdin Bio-Chem Technology Co., Ltd. (Shanghai, China). Hyaluronic acid (HA, MW 100–200 kDa) was purchased from Bloomage Freda Biopharm, Co., Ltd. (Shanghai, China). Singlet oxygen sensor green (SOSG) was purchased from Invitrogen (Carlsbad, CA, USA). Deionized water (Millipore) with a resistivity of 18 MΩ cm was used for all experiments. Dulbecco’s modified Eagle’s medium (DMEM), phosphate-buffered solution (PBS), and cell counting kit-8 (CCK-8) were bought from Nanjing Keygen Biotech. Co., Ltd. (Nanjing, China). Heat-inactivated fetal bovine serum (FBS) was bought from Gibco Laboratories (NY, USA). MCF-7 cells were acquired from American Type Culture Collection (ATCC, Manassas, VA).

### Synthesis of Mesoporous Organosilica Nanospheres (MONs)

MONs were synthesized according to a method reported in our previous work [37]. Typically, we prepared a solution composed of 25–28 wt% ammonia aqueous sol (1.0 mL), ethanol (30 mL), a concentrated CTAB (0.16 g), and water (75 mL). Next, mixed organosilica precursors comprising TEOS (0.25 mL) and TESPTS (0.1 mL) were added quickly to the mixture. The mixture was stirred at 35 °C for 24 h and then centrifuged resulting in the formation of thioether-bridged MONs, then rinsed thrice with ethanol.

### Modifying Amino Groups

To graft amino groups on the MONs, the disulfide bonds that formed the thioether-bridged MONs were transformed to thiol groups through a reduction reaction and then covalently linked with NH_2_-maleimide. Briefly, 0.1 g of the MONs was dissolved in a mixture of 1.2 mL water, 4.4 mL dioxane, 0.20 g triphenylphosphine, and 80 μg HCl solution (37 wt%). After heating to 40 °C in an atmosphere of nitrogen for 2 h, the thiol group linking the MONs was obtained by centrifugation of the other mixed solution, and washed thrice with water. Subsequently, the precipitation was dispersed in mixture of 15 mL water, 15 mL *N*,*N*-dimethylformamide as well as 3.24 mg NH_2_-maleimide. After shaking for 12 h at room temperature, amino-modified MONs (denoted as MONs-NH_2_) were obtained by washing with water for three times.

### Preparation of MONs-HA and SMONs-HA

MONs-HA were synthesized by coupling the MONs-NH_2_ with carboxyl groups activated HA. Briefly, 0.1 g HA was first dissolved in 5 mL water. After adding NHS (20 mg mL^−1^, 2.5 mL) and EDC (20 mg mL^−1^, 2.5 mL), this preparation was incubated at room temperature (RT) for 3 h while shaking. Thus, the carboxyl groups of HA were activated. Next, 0.08 g of MONs-NH_2_ was mixed with the solution containing carboxyl groups activated HA, and the mixture was shaken for 24 h. Then HA-modified MONs (denoted as MONs-HA) were obtained by centrifugation and washing thrice with water. Finally, the MONs-HA product without CTAB templates was obtained via three solvent extractions in ethanol (300 mL) at 27 °C for 12 h. To obtain SMONs-HA, the MONs-HA (2 mg mL^−1^) were dispersed in 0.1 M NaOH aqueous solution for 15 min. After washing with water, it was mixed with ethanol and stored until use.

### Preparation of MONs-HA-Cy5.5 and SMONs-HA-Cy5.5

In brief, the MONs-HA (60 mg) and SMONs-HA (20 mg) with the same number of particles (etched from the 60 mg of MONs-HA) were, respectively, dispersed in a mixture (water (12 mL) Cy5.5-maleimide (0.19 mg), *N*,*N*-dimethylformamide (1.2 mL)). Following shaking for 12 h at RT, Cy5.5-modified nanoparticles were obtained (denoted as MONs-HA-Cy5.5 and SMONs-HA-Cy5.5, in the order) by centrifuging and washing with water.

### Preparation of SMONs-HA-Ce6 and MONs-HA-Ce6

The carboxyl groups of Ce6 were activated by mixing Ce6 (1 mg mL^−1^ in *N*,*N*-dimethylformamide, 0.5 mL), NHS (1 mg), and EDC (1 mg) under shaking at RT for 3 h. Then, 1 mg of the MONs-HA-Cy5.5 or SMONs-HA-Cy5.5 was added. After shaking for 24 h, the Ce6 grafted nanoparticles (denoted as MONs-HA-Ce6 and SMONs-HA-Ce6, respectively) were obtained by centrifuging and washing with water.

### Characterization

A transmission electron microscope (TEM) was utilized to characterize the nanoparticles, with operation settings comprising 100 kV on a Hitachi HT7700 microscope (Tokyo, Japan). The electron microscope (FEI Talos F200X) equipped with an energy-dispersive X-ray (EDX) was employed to obtain high-resolution transmission electron data and elemental mapping. Scanning electron microscopy (SEM) measurements were performed at 5 kV (Hitachi S4800 microscope from Tokyo, Japan). Hydrodynamic size and zeta potential were measured using a ZetaPALS analyzer (Brookhaven Instruments Co., Holtsville, USA). An NEXUS870 spectrometer was employed to obtain Fourier transform infrared (FTIR) spectra. The Bruker AVIII400 spectrometer (9.47 T, 79.48 MHz of operating frequency, 6.0 kHz of spin rate, and 120 s of recycle delay) was utilized to obtain the ^29^Si magic-angle spinning (MAS) NMR spectra. A Micromeritics ASAP analyzer (2020) was employed to determine the Nitrogen adsorption–desorption isotherms at − 196 °C. First, the materials were degassed at 150 °C under vacuum for 10 h or more. By using the adsorption data in a relative pressure (*p*/*p*_0_) range of 0.12 to 0.20, we calculated the specific surface area (SBET) using the Brunauer–Emmett–Teller (BET) approach. We employed suitable nonlocal density functional theory (NLDFT) approaches to acquire pore-size distribution. Utilizing the amount adsorbed at a *p*/*p*_0_ of 0.994, we estimated the total pore volume. Young’s modulus of the samples was estimated with the AFM in the mode of Peak Force Quantitative Nanomechanical Mapping (PeakForce QNM, Dimension Icon AFM, Bruker, U.S.A.) employing the TESPA-V2 AFM cantilever (Bruker AFM probes). After placing a drop of aqueous sample suspension on a clean silicon surface, the Hertzian model was used to calculate Young’s modulus according to the slope in the linear region of the retraction curve. Infinite M200 PRO microplate scanner (Tecan, Switzerland) was used to record the intensity of the fluorescence.

### Cytotoxicity Assessment

The cytotoxic MCF-7 cells in a Complete Medium DMEM solution and then cultured in a 96-well plate (10,000 cells in each well) for 12 h. Next, we removed the supernatant and grown cells with 200 μL of MONs-HA-Cy5.5 or SMONs-HA-Cy5.5 at various concentrations (25–400 μg mL^−1^) for 24 h. Then, 20 μL of the CCK-8 solution was mixed. Finally, the absorbance (OD_450nm_) was read after 1 h with a microplate scanner. Cell viability (CV) was estimated as follows:

$$ {\text{CV}} = \frac{{A_{\text{sample}} - A_{{( - ){\text{control}}}} }}{{A_{{( + ){\text{control}}}} - A_{{( - ){\text{control}}}} }}. $$

### Hemocompatibility Assay

The human blood sample was donated by a healthy young woman with the approval of the local medical ethics committee. In brief, 10 mL of normal saline (NS) were mixed with blood samples (2 mL), and centrifuged (2000 rpm, 5 min) to isolate the red blood cells (RBC). The RBCs were washed using NS for several times. When the supernatant turned colorless, the RBCs were resuspended in 10 mL of NS. A mixture of 200 μL of RBCs and 800 μL of the MONs-HA-Cy5.5 or SMONs-HA-Cy5.5 with different concentrations (25–400 μg mL^−1^) was prepared. To set negative or positive control, (200 μL) 800 μL of NS or water was, respectively, mixed with RBC suspension. After maintaining at 37 °C for 2 h, the mixtures were centrifuged to get supernatant. A microplate reader was used to measure the concentration at OD of 570 nm. Hemolysis (H) was determined as follows:

$$ {\text{H}} = \frac{{{\text{OD}}_{\text{sample}} - {\text{OD}}_{{( - ){\text{control}}}} }}{{{\text{OD}}_{{( + ){\text{control}}}} - {\text{OD}}_{{( - ){\text{control}}}} }}. $$

### In Vivo Toxicity

MONs-HA-Cy5.5 (100 μL) and SMONs-HA-Cy5.5 (100 μL) were injected respectively into male ICR mice (age; 4 weeks; *n* = 3; 20 mg kg^−1^). The mice were killed 21 days post-injection for harvesting of key organs (heart, liver, spleen, kidney, and lungs). Subsequently, the organs were subjected to hematoxylin and eosin (H&E) staining assay. Finally, the sections were imaged using an optical microscope (IX71; Olympus, Tokyo, Japan).

### Cellular Uptake

Human breast cancer MCF-7 cells were grown in 6-well plates (1 × 10^6^ cells per well) overnight. Subsequently, we substituted the medium in every well with 2 mL of fresh growth medium comprising MONs-HA-Cy5.5 or SMONs-HA-Cy5.5. The final concentration of MONs-HA-Cy5.5 and SMONs-HA-Cy5.5 were 100 and 33 μg mL^−1^, respectively. The 33 μg mL^−1^ of SMONs-HA-Cy5.5 was obtained from 100 μg mL^−1^ of MONs-HA-Cy5.5 by etching approach, and thus they have the same number of particles. After culturing the cells for 1, 3, and 6 h, they were digested and suspended in 200 μL of PBS after washing with PBS for three times. The flow cytometry system was employed for quantification of cellular uptake of the two nanoplatforms. Six hours following incubation with MONs-HA-Cy5.5/SMONs-HA-Cy5.5, the cells were fixed for 20 min with 4% paraformaldehyde, then stained with DAPI. The intracellular association and nuclear morphology of the nanoplatforms were examined using confocal laser scanning microscopy (CLSM). The fluorescence intensity was analyzed using image-J software.

### In Vivo Blood Circulation

To evaluate the in vivo circulation behavior of the MONs-HA-Cy5.5 and SMONs-HA-Cy5.5, BALB/c mice were injected with MONs-HA-Cy5.5 or SMONs-HA-Cy5.5 (100 μL, 20 mg kg^−1^) through the tail vein. Then the blood sample (20 µL) was collected at different time points (1, 3, 5, 10, 15, 30 min, and 1, 3, 6 h) after injection. The blood was added into the anticoagulant tube, which contains NS (1 mL). Inductively coupled plasma (ICP) was used to measure the Si concentration. In vivo terminal half-life and other pharmacokinetic parameters of the MONs-HA-Cy5.5 or SMONs-HA-Cy5.5 was determined by a double-component pharmacokinetic model using DAS 3.0 (BioGuider Co., Shanghai, China).

### In Vivo Biodistribution

MCF-7 tumors were subcutaneously injected (2 × 10^7^ tumor cells) in the right forelimb of female BALB/c nude mice (age; 6 weeks). The amounts of the tumor in mice were checked daily until a volume of 200 mm^3^ was reached. To further identify the difference between the MONs-HA-Cy5.5 and SMONs-HA-Cy5.5 in terms of in vivo biodistribution, MONs-HA-Cy5.5 (100 μL, 20 mg kg^−1^) or SMONs-HA-Cy5.5 (100 μL, 6.67 mg kg^−1^) with the same number of particles were administered intravenously into MCF-7 tumor-bearing BALB/c mice (*n* = 3). Twenty-four hours after the treatment, we killed the mice and harvested the tumors plus major organs, which included: heart, spleen, liver, kidney, lung, and muscle tissues flanking the tumors. An IVIS Lumina XR system under a Cy5.5 filter (*λ*_ex_ = 640 nm, *λ*_em_ = 705 nm) was employed to image the harvested specimens. To further investigate the long-term accumulating effect of the samples in each organ, the mice (*n* = 3) were killed at different times (including 3, 6, 12, 24, 48, and 72 h) post-injection. Subsequently, their major organs were harvested and imaged as described above.

### Penetration in MCF-7 Tumor Spheroids

To establish MCF-7 multicellular spheroids (MCSs) as an in vitro tumor model, MCF-7 cells grown in 96-well plates (10,000 cells per well] were precoated by 50 μL of 1.5 w/v% hot agarose solution and then cooled to RT) and cultured for 4 days at 37 °C to grow into spheroids. Then the MCSs were co-cultured with the MONs-HA-Cy5.5 (100 μg mL^−1^) or SMONs-HA-Cy5.5 (33 μg mL^−1^) with the same number of particles. Four hours later, the MCSs were harvested and rinsed with PBS to be examined using CLSM. The analysis of images was conducted using image-J.

### Intratumoral Distribution

To study the intratumoral penetration of the MONs-HA-Cy5.5 and SMONs-HA-Cy5.5, mice bearing MCF-7 xenografts were intravenously injected with MONs-HA-Cy5.5 (100 μL, 20 mg kg^−1^) or SMONs-HA-Cy5.5 (100 μL, 6.67 mg kg^−1^) with the same number of particles. The mice were killed 24 h later to extract the tumors, which were sectioned into slides of 8 µm thickness in a cryostat. We stained the blood vessels following a standard protocol [38], and the tumor slices were then observed using CLSM.

### Detection of Reactive ^1^O_2_

Singlet oxygen was detected using SOSG. The intrinsic fluorescence of SOSG increases in the presence of ^1^O_2_. Briefly, MONs-HA-Ce6 or SMONs-HA-Ce6 (100 µL, 1 × 10^−6^ m Ce6 equiv.) were mixed with SOSG (10 µL, 50 × 10^−6^ M) in H_2_O_2_ solution (3 wt%). Then, the mixture was irradiated by a 660-nm laser (0.5 W cm^−2^) for different time (0, 1, 3, 5, 10 min). Finally, a microplate reader (*λ*_ex_ = 490 nm, *λ*_em_ = 530 nm) was used to measure the intensity of the fluorescence.

### Determining Reactive Oxygen Species (ROS) in Cells

DCFH-DA was employed to evaluate the accumulation of total ROS in the cells. The total ROS was indicated by fluorescence intensity adducts of DCFH-DA [i.e., dichlorofluorescein (DCF)]. Briefly, MCF-7 cells (1 × 10^5^ cells/well) were incubated in 96-well plate in DMEM (100 µL) supplemented with 10% FBS and cultured overnight. Next, we co-incubated the cells with Ce6, MONs-HA-Ce6, or SMONs-HA-Ce6 (100 µL, 4 × 10^−6^ M Ce6 equiv.) for 6 h. The control group was set by adding 100 µL of PBS. The media were then replaced by fresh DMEM (100 µL) supplemented with DCFH-DA (20 × 10^−6^ M) followed by a further 4 h incubation. Next, we washed the cells with PBS and subjected them to 660-nm laser irradiation (0.5 W cm^−2^) for 5 min. Finally, a microplate reader (*λ*_ex_ = 485 nm, *λ*_em_ = 525 nm) was employed to record the fluorescence intensity.

### In Vitro PDT

MCF-7 cells (1 × 10^5^ cells/well) were seeded into a 96-well plate and cultured for 12 h. After that, we co-incubated the cells for 24 h with 200 μL of Ce6, MONs-HA-Ce6, or SMONs-HA-Ce6 at various Ce6 concentrations (1–4 × 10^−6^ M Ce6 equiv.). Next, the cells were subjected to a 660-nm laser (0.5 W cm^−2^) radiation for 5 min. The experiments were repeated three times. The therapeutic effects were analyzed using the CCK8 assay.

### In Vivo Antitumor Effect

BALB/c mice inoculated with MCF-7 tumors (*n* = 5, each group) were grouped into seven groups: (I) PBS group, (II) MONs-HA-Ce6 group, (III) SMONs-HA-Ce6 group, (IV) PBS + laser group, (V) free Ce6 + laser group, (VI) MONs-HA-Ce6 + laser group, (VII) SMONs-HA-Ce6 + laser group. Subsequently, we injected 100 μL of each material intravenously (The relevant dosage of Ce6 of each group was approximately 6 mg kg^−1^ except the PBS group). Twenty-four hours following injection, we subjected the mice to 660-nm laser radiation (0.5 W cm^−2^, 10 min). Four mice per group were killed at 24 h post-irradiation, and the collected tumors were stained with hematoxylin and eosin (H&E) for histopathology assessment. We recorded the tumor volumes and body weights for 14 days consecutively. The equation below was used to calculate the tumor volume (V): V = Width^2^ × length/2. The volume of tumor after therapy was divided by the volume before therapy to obtain the relative tumor volume (*V*/*V*_0_; *V*_0_ is tumor volume pretherapy). On the last day of the experiment, we killed the mice and took photographs and weights of the tumors. Approval for conducting these experiments was obtained from the Animal Care and Use Committee of Jinling Hospital.

### Data Analysis

Experimental were processed using GraphPad Software (CA, USA) and are shown as mean ± standard deviation. Results of two groups were compared using student’s *t* test, whereas multiple groups were compared using the variance (ANOVA) test. Significance was set a *P* < 0.05.

## Results and Discussion

### Characterization of SMONs-HA-Cy5.5 and MONs-HA-Cy5.5

**Fig. 1:**
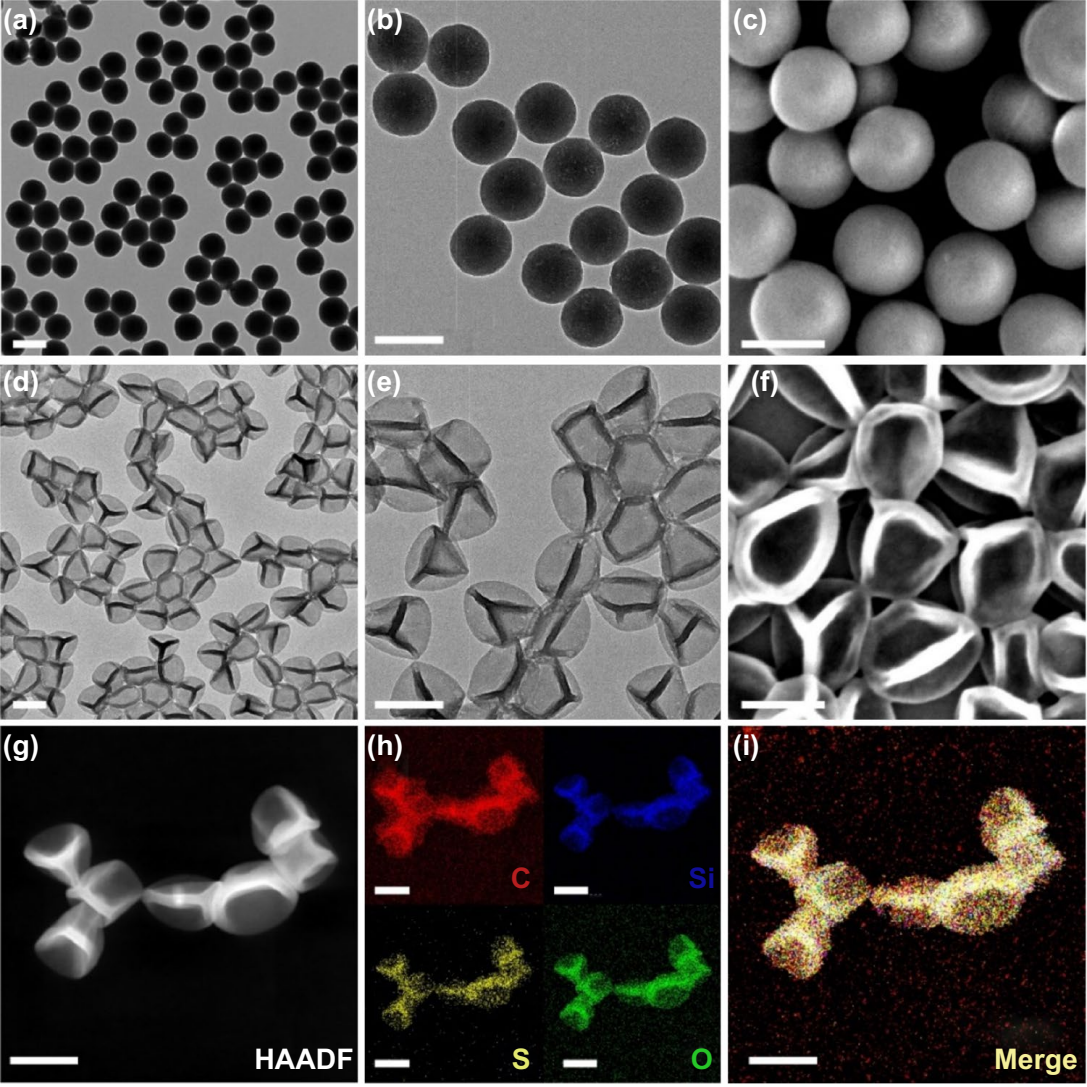
**a**, **b** TEM and **c** SEM images of the MONs-HA-Cy5.5. **d**, **e** TEM and **f** SEM images of the SMONs-HA-Cy5.5. **g** STEM-HAADF image and **h**, **i** EDX elemental mapping images of the SMONs-HA-Cy5.5. Red, blue, yellow, and green indicate the elements C, Si, S, and O, respectively. Scale bars, 200 nm. (Color figure online)

The CTAB-directed solgel process was utilized to prepare thioether-bridged MONs and then covalently modified with HA and Cy5.5 after reducing thioether moieties and connecting with NH_2_-maleimide. The TEM and SEM images indicated that both the MONs and MONs-HA-Cy5.5 possess a well-defined spherical shape with an identically uniform diameter of 180 nm (Figs. 1a–c and S1). The soft SMONs-HA were prepared using a preferential etching approach. After being etched in an aqueous NaOH solution (0.1 M) for 15 min, the surface of the MONs-HA collapsed and uniformly shrank inward, transforming into a cross-wrinkled morphology. According to the SEN and TEM images, the SMONs-HA-Cy5.5 have a hollow structure (Fig. 1d–f). The shell thickness, diameter, and cavity size of the SMONs-HA-Cy5.5 are estimated to be 24, 205, and 160 nm, respectively. STEM-HAADF images further confirm the symmetrically cross-wrinkled morphology and the ultrathin shell (Fig. 1g). EDX elemental mapping of the SMONs-HA-Cy5.5 indicated that C, Si, S, and O are mainly distributed on the outside ridge of the hollow structure, while C, Si, S, and O are homogeneously deposit in the whole solid sphere before etching (Figs. 1h–i and S2). The mechanism of preparing SMONs from MONs has been reported in our previous work. Directly speaking, the organosilica nanospheres have a more stable surface with highly cross-linked Si–OH groups via ammonia catalysis in the sol–gel process directed by CTAB. The less condensed cores of the MONs-HA and the inorganic silica species in the shell are preferentially etched away by OH^−^ ions when dispersed in NaOH solution, resulting in significantly increased ratio of flexible Si–R–Si chains, and noncross-linked free Si–OX groups in holistic frameworks. The shell becomes thinner and deform inward via internal van der Waals force, which eventually results in the cross-linked and coarse morphology of SMONs-HA. More importantly, the dominating free Si–OX groups in incomplete condensation and flexible Si–R–Si chains, as well as the hollow nanostructure with ultrathin shells amid the frameworks, grant SMONs-HA the unique soft character.

**Fig. 2:**
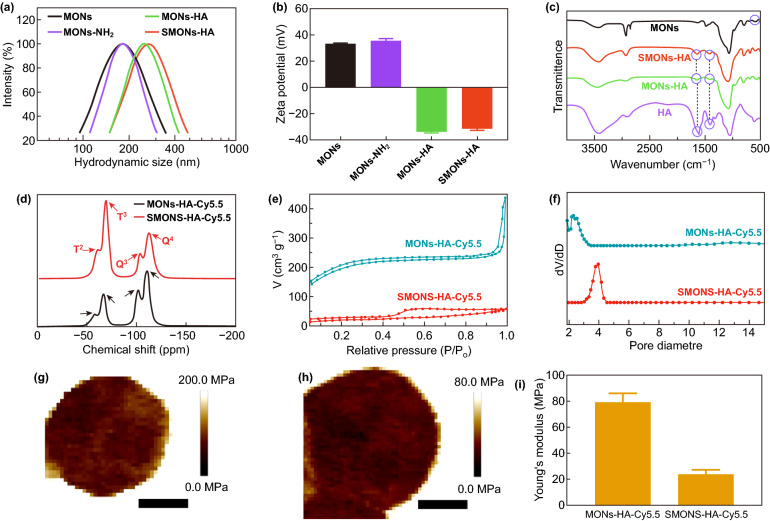
**a** Hydrodynamic diameter and **b** zeta potential of the MONs, MONs-NH_2_, MONs-HA, and SMONs-HA. **c** FTIR spectrum of the MONs, HA, MONs-HA, and SMONs-HA. **d** ^29^Si MAS NMR spectra, **e** nitrogen sorption isotherms, **f** pore-size distribution curves, **g**, **h** AFM images and **i** Young’s modulus of the MONs-HA-Cy5.5 and SMONs-HA-Cy5.5. Scale bars, 100 nm

The hydrodynamic diameter, zeta potential, chemical composition, and mesoporous structure of the soft SMONs-HA and the hard counterparts were evaluated. The hydrodynamic diameters of the MONs, MONs-NH_2_, MONs-HA and SMONs-HA are measured to be about 181, 184, 251, and 268 nm, respectively (Fig. 2a). Both the MONs-HA and SMONs-HA have a narrow polydispersity index (PDI): 0.106 ± 0.01 and 0.139 ± 0.02, suggesting their excellent dispersity in aqueous solutions. The zeta potential of the MONs, MONs-NH_2_, MONs-HA and SMONs-HA are measured to be 33.0 ± 0.2, 35.4 ± 1.7, − 33.8 ± 0.6, and − 31.4 ± 1.2 mV, respectively (Fig. 2b). The negatively charged surfaces for the MONs-HA and SMONs-HA demonstrate the successful modification of HA. Notably, there is no significant fluctuation in surface charge after the MONs-HA were etched, suggesting the stability of the modified HA. Furthermore, the FTIR spectrum of the MONs exhibits C–S absorbance band at 691 cm^−1^, indicating the thioether group-incorporated frameworks (Fig. 2c) [39]. Clearly, both SMONs-HA and MONs-HA show the absorbance bands at 1630 and 1380 cm^−1^ which are assigned to the carboxylate asymmetric and symmetric stretching vibration of the HA skeleton, respectively [40]. These results clearly illustrate the successful modification of HA on both MONs-HA and SMONs-HA. Furthermore, to make the materials detectable, a near-infrared fluorescent dye Cy5.5 was modified on the nanoplatforms, and the fluorescence intensities of the soft SMONs-HA-Cy5.5 and MONs-HA-Cy5.5 were modulated to similar values (Fig. S3). The 29Si MAS NMR spectra of SMONs-HA-Cy5.5 and MONs-HA-Cy5.5 both exhibited signals at − 110, − 58, − 102, and − 67 ppm, respective to *Q*^4^ (Si(OSi)_4_), *Q*^3^ (Si(OSi)_3_(OX)), *T*^3^ (C–Si(OSi)_3_), and *T*^2^ (C–Si(OSi)_2_(OX)) species, in that order (Fig. 2d). The presence of the *T* peak reveals the organic group-incorporated frameworks. The *T*^*n*^/(*T*^*n*^ + *Q*^*m*^) ratio of the SMONs-HA-Cy5.5 is 0.604, which is remarkably higher in comparison with the MONs-HA-Cy5.5 (0.326), demonstrating that Q silicate species in frameworks are preferentially etched in NaOH aqueous solution. Relatively higher organic group content remained in the frameworks of SMONs-HA-Cy5.5 are favorable to the soft structure and deformability. The nitrogen sorption isotherms of the SMONs-HA-Cy5.5 and MONs-HA-Cy5.5 were further measured, which show type IV curves (Fig. 2e), indicating typical mesoporous structures. The comparatively large hysteresis loop of the SMONs-HA-Cy5.5 suggests the appearance of hollow cavities. The surface areas of the SMONs-HA-Cy5.5 and MONs-HA-Cy5.5 are estimated to be 80.8, and 495.6 m^2^ g^−1^, respectively, and their pore volumes are 0.086 and 0.416 cm^3^ g^−1^, respectively. Also, the pore sizes of the SMONs-HA-Cy5.5 and MONs-HA-Cy5.5 are calculated to be 4.0 nm and 2.3 nm based on the NLDFT method, respectively (Fig. 2f). Furthermore, the elastic Young’s modulus (*E*_y_) of the SMONs-HA-Cy5.5 is measured to be 24.2 ± 3.2 MPa by using atomic force microscopy (AFM), which is about 31% of the *E*_y_ of their hard counterparts (79.2 ± 7.0 MPa, Fig. 2g–i), clearly certifying the SMONs-HA-Cy5.5 are significantly “softer” than the MONs-HA-Cy5.5. Additionally, the diameter of the SMONs-HA-Cy5.5 is slightly larger than that of MONs-HA-Cy5.5 when observed using AFM, which is most likely because that the SMONs-HA-Cy5.5 were squashed more significantly under compression during the testing process owing to its softer characteristic comparing with the hard ones. These results indicate that soft mesoporous organosilica nanoplatforms SMONs-HA-Cy5.5 and the hard MONs-HA-Cy5.5 with similar size, surface charge, component, fluorescence intensity have been obtained successfully. The hydrodynamic diameters of SMONs-HA-Cy5.5 and MONs-HA-Cy5.5 remain notably stable after storing in different solvent conditions for 2 weeks, indicating these nanoparticles are remarkably steady (Table S1). The hydrodynamic diameters and zeta potential of MONs-HA-Cy5.5 and SMONs-HA-Cy5.5 are measured to be 237.8, 249.2, − 15.6, and − 12.5 mV after been stored in PBS aqueous solution for 2 weeks (Table S1 and Fig. S4). The value of zeta potential in PBS is lower than that in water, which is mainly because that the counter ions in PBS solution accumulate on the surface of nanoparticle, shielding the surface charge and thus reducing the absolute value of zeta potential. But the hydrodynamic diameters of MONs-HA-Cy5.5 and SMONs-HA-Cy5.5 measured in PBS solution stay similar with that measured in water due to the sterically stabilizing effect of HA. The results indicate that MONs-HA-Cy5.5 and SMONs-HA-Cy5.5 could be stored in PBS aqueous solution for at least 2 weeks and keep excellent dispersity.

### Biocompatibility

To qualify the following biomedical applications, the cytotoxicity of the SMONs-HA-Cy5.5 and MONs-HA-Cy5.5 to MCF-7 cells were examined. The cell viabilities remain over 80% even treated with SMONs-HA-Cy5.5 or MONs-HA-Cy5.5 at a concentration of up to 400 μg mL^−1^ for 24 h, and the hemolytic activities of both the SMONs-HA-Cy5.5 and MONs-HA-Cy5.5 are lower than 1% at a concentration of up to 400 μg mL^−1^, indicating excellent hemocompatibility (Fig. S5). The in vivo biocompatibility was further evaluated through hematoxylin and eosin (H&E) staining, blood routine, and serum biochemical examinations. At 21 days after intravenous injection of the SMONs-HA-Cy5.5 or MONs-HA-Cy5.5 at the dosages of 20 mg kg^−1^, the complete blood counts and serum chemistries show that no significant changes happen compared with normal saline injected controls. All parameters including blood cells (WBCs), red blood cells (RBCs), platelet (PLT), alanine aminotransferase (ALT), aspartate aminotransferase (AST), alkaline phosphatase (ALP), uric acid (UA), and direct bilirubin (D-BLL) are within normal reference ranges (Fig. S6a–h), indicating problems like myelosuppression, hemolysis, inflammation, or liver and kidney injury will not be caused by intravenous injection of the materials. Besides, there is no obvious inflammation or necrosis of major organs (heart, liver, spleen, lung, and kidney) in the H&E staining images (Fig. S6i), which further indicates the excellent in vivo safety of the SMONs-HA-Cy5.5 and MONs-HA-Cy5.5.

### In Vitro Cellular Uptake

**Fig. 3:**
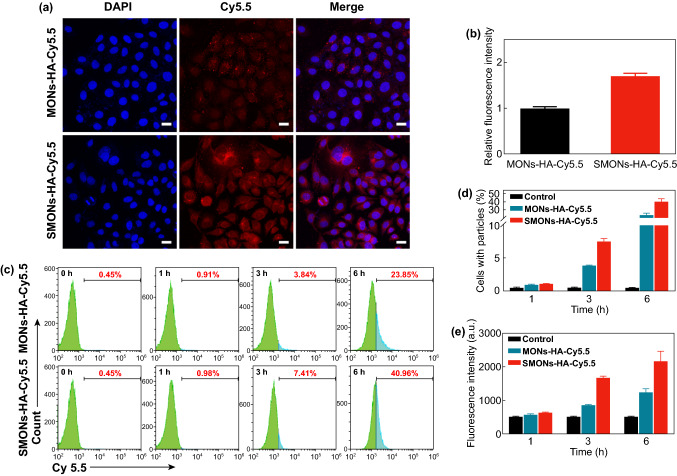
Cellular uptake. **a** CLSM images and **b** relative fluorescence intensity analysis of MCF-7 cells incubated with the MONs-HA-Cy5.5 and SMONs-HA-Cy5.5 for 6 h. Scale bars, 25 µm. **c** Flow cytometry, **d** relative cellular uptake efficiency and **e** mean fluorescence intensity of MCF-7 cells incubated with the MONs-HA-Cy5.5 and SMONs-HA-Cy5.5 for 1, 3, 6 h

The tumor cellular uptake of the SMONs-HA-Cy5.5 and MONs-HA-Cy5.5 was investigated using MCF-7 cells. The CLSM images show that the fluorescence signals are significantly stronger when the cells were incubated with the SMONs-HA-Cy5.5 (Figs. 3a and S7,). The relative intracellular Cy5.5 fluorescence intensity of MCF-7 cells incubated with the SMONs-HA-Cy5.5 for 3 or 6 h is about 1.7 times higher than that of MONs-HA-Cy5.5 group (Figs. 3b and S8). Furthermore, the cellular uptake of the SMONs-HA-Cy5.5 and the MONs-HA-Cy5.5 was quantitatively investigated using flow cytometry. The percentages of cells associated with MONs-HA-Cy5.5 are measured to be 0.91%, 3.84%, and 23.85% after incubation with MONs-HA-Cy5.5 for 1, 3, and 6 h, respectively (Fig. 3c, d). As for SMONs-HA-Cy5.5 group, the numbers increase to 0.98%, 7.41%, and 40.96% at the matched time intervals. The cellular uptake of SMONs-HA-Cy5.5 is almost twice that of their stiff counterparts after incubation with MCF-7 cell for 6 h. In addition, the relative average fluorescence intensities of SMONs-HA-Cy5.5 treated group are measured to be 1668 and 2159 after incubation for 3 and 6 h (Fig. 3e), both of which are up to ~ 2-fold higher than that of the rigid MONs-HA-Cy5.5 group (852 and 1237). These findings strongly certify that the soft SMONs-HA-Cy5.5 nanoplatforms are more efficiently internalized by MCF-7 cells than the stiff ones. It is believed that the deformability of soft nanoparticles can decrease the invagination of cellular membranes and thus save the corresponding energy needed for the wrapping of nanoparticles during internalization [37].

### Blood Circulation

**Fig. 4:**
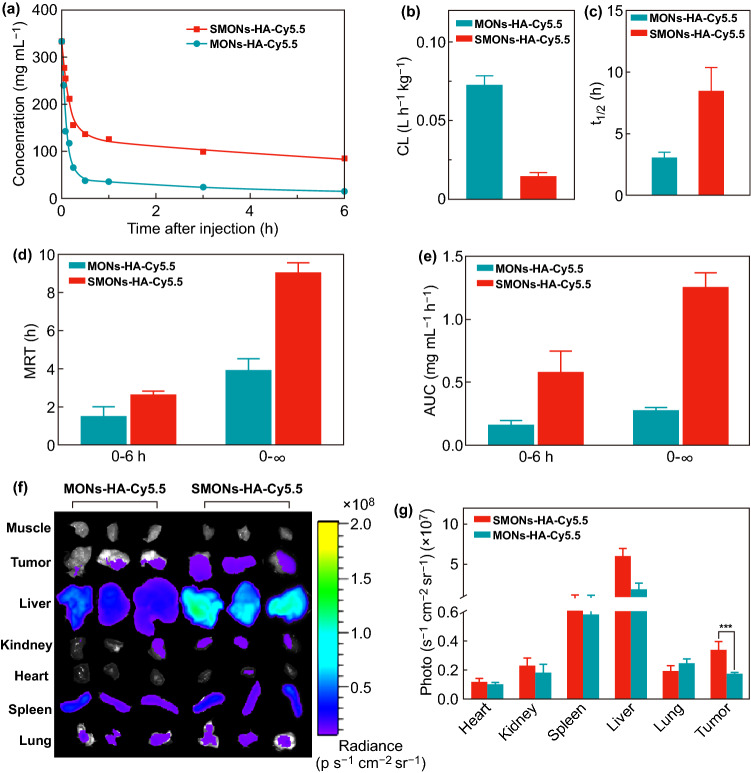
Blood circulation and tumor accumulation. **a** Concentrations of Si in the blood at different time intervals after injection of MONs-HA-Cy5.5 and SMONs-HA-Cy5.5. **b** The corresponding clearance (CL), **c** the terminal phase half-life(*t*_1/2_), **d** the mean residence time (MRT) and **e** blood concentration versus time curve (AUC) of MONs-HA-Cy5.5 and SMONs-HA-Cy5.5 by fitting the circulation profile. **f** Near-infrared fluorescence (NIFR) imaging and **g** quantification of the fluorescence intensity in the major organ and tumor from MCF-7 bearing mice (*n* = 3) at post-24 h after the administration of the SMONs-HA-Cy5.5 or MONs-HA-Cy5.5. ***P < 0.001

The in vivo application of nanoparticle (NP)-based drugs has been limited by their short circulation half-life [41]. To explore whether the soft nanoplatforms have advantages in pharmacokinetics, the SMONs-HA-Cy5.5 and MONs-HA-Cy5.5 were separately injected into ICR mouse via tail vein. The circulation profiles were then obtained by measuring the Si concentration in blood samples (Fig. 4a). There is a sharp decline in the first 0.5 h after dosing in the concentration–time curves, indicating a distribution phase: the concentrations of SMONs-HA-Cy5.5 and MONs-HA-Cy5.5 drop to 41.1% and 11.5% of injection dose, respectively. In the late elimination phase, the SMONs-HA-Cy5.5 group exhibits approximately 5-fold higher blood drug concentration compared with the MONs-HA-Cy5.5 group. At 6 h after intravenous injection, the remaining levels of SMONs-HA-Cy5.5 and MONs-HA-Cy5.5 in blood are measured to be 25.5% and 4.7%. The pharmacokinetic analysis demonstrates that the corresponding clearance (CL) is 0.0143 for the SMONs-HA-Cy5.5 and 0.0727 L h^−1^ kg^−1^ for MONs-HA-Cy5.5 (Fig. 4b). The significantly decreased CL for the SMONs-HA-Cy5.5 indicates that the soft nanoplatforms provide a slower clearance from the circulation and a prolonged blood retention. Additionally, the terminal phase half-life of the SMONs-HA-Cy5.5 is 8.49 h (Fig. 4c), nearly 3 times longer than that of MONs-HA-Cy5.5 (3.07 h). Furthermore, the mean residence time (MRT) and correlative area under the blood concentration versus time curve (AUC) were also calculated using a two-compartment model. MRT_0–6h_ and MRT_0–∞_ of the SMONs-HA-Cy5.5 are 3.94 and 9.09 h, whereas the numbers for MONs-HA-Cy5.5 are 1.45 and 2.67 h (Fig. 4d). AUC_0–6h_ and AUC_0–∞_ of the SMONs-HA-Cy5.5 are 0.582 and 1.258 mg mL^−1^ h^−1^, while the corresponding values for the MONs-HA-Cy5.5 are 0.165 and 0.276 mg mL^−1^ h^−1^ (Fig. 4e). Notably, the SMONs-HA-Cy5.5 show significant advantages (3–4 fold) over the stiff counterparts in terms of these pharmacokinetic parameters. These results clearly demonstrate that the soft nanoplatforms display a longer blood circulation in vivo than the hard ones. The possible explanation for the prolonged circulation time of deformable nanoplatforms is that they have unique fluid mechanics compared to stiff ones in the flowing bloodstream [42, 43].

### In Vivo Biodistribution

Since long circulation time in vivo is strikingly essential for nanomaterials to accumulate in solid tumors via the EPR effect, the accumulation of the SMONs-HA-Cy5.5 and MONs-HA-Cy5.5 in tumors was further evaluated in MCF-7 tumor-bearing BALB/c nude mice. The fluorescence intensities of the SMONs-HA-Cy5.5 and MONs-HA-Cy5.5 solutions were very close (Fig. S9). At 24 h post-injection, the mice were killed, and their tumors and major organs were collected and subjected to the IVIS Lumina *XR* system. Notably, the tumors of the mice injected with the MONs-HA-Cy5.5 show stronger fluorescence than those injected with MONs-HA-Cy5.5 (Fig. 4f). The average fluorescence intensity of the tumors in the SMONs-HA-Cy5.5 group is 0.34 × 10^7^ photo s^−1^ cm^−2^ sr^−1^, twice higher than that of MONs-HA-Cy5.5 group (0.17 × 10^7^ photo s^−1^ cm^−2^ sr^−1^) at 24 h after intravenous injection (Fig. 4g). These results demonstrate that tumor accumulation of soft SMONs-HA-Cy5.5 is significantly higher than that of the rigid counterparts, indicating that the soft structure of the SMONs-HA-Cy5.5 is indeed conducive to their accumulation in tumor sites, which is related to its prolonged circulation time and more efficient cellular uptake. It is noted that although the accumulation of SMONs-HA-Cy5.5 in tumors is higher than that of MONs-HA-Cy5.5, both of them are still low considering the accumulation in the liver or spleen. It is due to the fact that the liver and spleen are major biological barriers that prevent drug delivery to solid tumors because they sequester the majority of administered nanomaterials [44, 45]. Thus, more elaborated modifications are needed in their future application. For example, coat the soft nanoplatforms with a dense and dynamic outer PEG layer or cell-membrane components to cloak the particles from liver endothelial cells [15, 16], modify tumor-targeting ligands on the surface of nanoparticles to further enhance their accumulation in tumors [42, 46]. A series of ex vivo images of mice over 72 h show that fluorescence signals are mainly concentrated in the liver and spleen, and the intensity of fluorescence signals decrease gradually with the prolongation of injection time (Fig. S10). The results indicate that most of the nanoparticles are engulfed by the reticuloendothelial system and metabolized through the liver and spleen. Interestingly, during the initial period after injection, the soft SMONs-HA-Cy5.5 accumulate in the liver and spleen more rapidly than MONs-HA-Cy5.5. However, with the time going on, the accumulation of liver and spleen of soft SMONs-HA-Cy5.5 group and MONs-HA-Cy5.5 tends to be the similar after 24 h post-injection.

### Tumor Penetration in Multicellular Spheroids (MCSs)

**Fig. 5:**
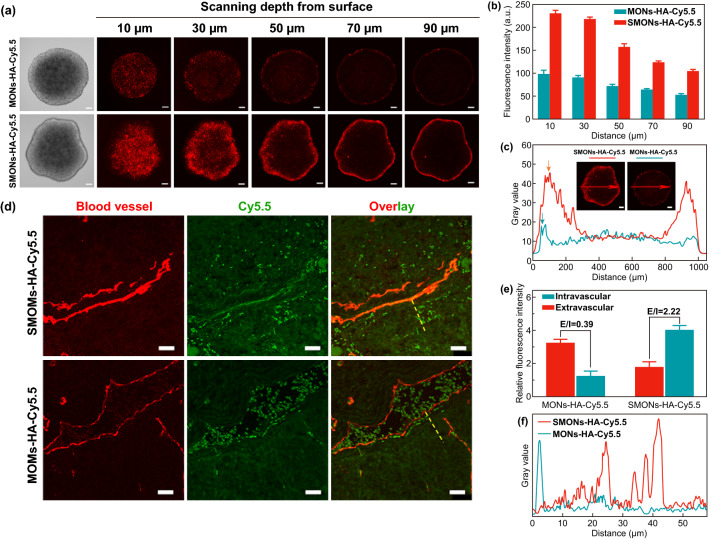
Penetrating behavior. **a** CLSM Z-stack scanning images and **b** corresponding fluorescence intensity of MCF-7 multicellular spheroids (MCSs) upon 4 h incubation with the MONs-HA-Cy5.5 and SMONs-HA-Cy5.5. Scale bars, 100 µm. **c** Gray value profile of MCSs along the arrow region at the Z-axis distance of 50 µm. **d** CLSM images of tumor slices from mice at post-24 h injection. Red and green signals were from anti-CD31 stained blood vessels and Cy5.5, respectively. Scale bars, 25 µm. **e** Relative Cy5.5 fluorescence intensities of intravascular and extravascular areas. **f** The grey value profile of green signal of extravascular areas (indicated by the yellow dashed lines). (Color figure online)

It is speculated that soft nanoparticles could deform and squeeze into ECM when they contact the surface of tumor. Herein, multicellular spheroids derived from MCF-7 cells were employed as in vitro 3D models to investigate the discrepancy in tumor penetration capacity between the SMONs-HA-Cy5.5 and the stiff counterparts. After co-incubated with SMONs-HA-Cy5.5 or MONs-HA-Cy5.5 for 4 h, the MCSs were subjected to CLSM Z-stack scanning. The outer edge of MCSs was defined as 0 µm. The CLSM images show that the fluorescence signals are stronger and penetrate deeper through the distance from 30 to 90 µm in SMONs-HA-Cy5.5 treated group, whereas the fluorescence signals of the rigid MONs-HA-Cy5.5 group are relatively weaker and mainly distribute in the peripheral area of MCSs (Fig. 5a). For example, at the scanning depth of 30 µm, the fluorescence intensity of the core region of the MCS in MONs-HA-Cy5.5 group is as weak as 9.8 (Fig. S11). Whereas the intensity in the core region of the MCS in the SMONs-HA-Cy5.5 group is 17.2, nearly twice the fluorescence signal intensity of its rigid counterpart, implying the SMONs-HA-Cy5.5 can pass through ECM easier and diffuse deeper into the tumor tissues. The fluorescence intensities of all scanning slices were measured to evaluate the penetration performance of SMONs-HA-Cy5.5 and MONs-HA-Cy5.5 in MCSs. As indicated in Fig. 5b, SMONs-HA-Cy5.5 group display more than 2-fold higher fluorescence intensity than MONs-HA-Cy5.5 group throughout the distance of 10 to 90 µm, suggesting significantly improved tumor penetration ability of SMONs-HA-Cy5.5. In addition, the distributions of the two nanoplatforms in MCSs at the Z-axis distance of 50 µm were further analyzed by comparing the gray value of the central linear region (Fig. 5c, horizontal red arrow). For the stiff MONs-HA-Cy5.5, the highest gray value (marked by a vertical blue arrow) appears at X-axis distance of 74 µm. In comparison, the soft SMONs-HA-Cy5.5 reach their distribution climax (marked by a vertical red arrow) at 104 µm distance, clearly demonstrating that a 30 µm-deeper penetration is achieved in SMONs-HA-Cy5.5 group. Additionally, the grey value of MCS in SMONs-HA-Cy5.5 is always higher than that in MONs-HA-Cy5.5 group from the surface to 300 µm-distance, which is consistent with their higher cellular uptake and deeper penetration. These results clearly demonstrate that the soft SMONs-HA-Cy5.5 have tremendous advantage over the stiff ones in tumor penetration.

### Ex Vivo Intratumoral Distribution

To compare the ex vivo intratumoral distribution of the two nanoplatforms with different softness, the MCF-7 xenograft tumor-bearing mice were injected with SMONs-HA-Cy5.5 or MONs-HA-Cy5.5. After 24 h, the mice were killed and the obtained tumors were sectioned and stained with anti-CD31 antibody to visualize the blood vessels. As can be seen in the CLSM images (Fig. 5d), the green fluorescence signal of Cy5.5 scatters in the tumor interstitium away from the blood vessel in SMONs-HA-Cy5.5 treated group, indicating the soft nanoplatforms can extravasate into perivascular tumor regions and diffuse into deeper tumor tissues. However, for MONs-HA-Cy5.5 treated group, the green fluorescence signal is mainly colocalized with blood vessel or located near the vessel wall, suggesting the poor extravasation and limited penetration. Moreover, the relative Cy5.5 fluorescence intensities (FI) of three intravascular and three extravascular regions were quantitatively analyzed. The extravascular FI/intravascular FI ratio in SMONs-HA-Cy5.5 treated group is measured to be 2.22, while the value for MONs-HA-Cy5.5 group is only 0.39 (Fig. 5e), demonstrating the soft SMONs-HA-Cy5.5 nanoplatforms have better intratumoral extravascular penetration capability. The extravascular distribution of the two nanoplatforms were further evaluated by depicting gray value profile of green signal of an extravascular area (yellow dashed lines). The peak of gray value appears at distance of 2.72 μm in MONs-HA-Cy5.5 group (Fig. 5f), implying that the stiff nanoplatforms are confined to the vessel wall. On the contrary, the distribution of the SMONs-HA-Cy5.5 could be observed until 43 μm away from the blood vessel, indicating their superior penetration performance. These results demonstrate that soft nanoplatforms have significantly improved extravasation and penetration behavior in tumor tissues. It is speculated that the SMONs-HA-Cy5.5 nanoplatforms can pass through tumor vessel wall fenestrations more easily and diffuse within the tumor ECM more efficiently, which is associated with their soft structure and deformability. In contrast, the MONs-HA-Cy5.5 are constricted within or around the vessels due to their stiffness and non-deformability. The ability of soft nanoplatforms to shrink and deform in dense and confined environments can boost their diffusion compared to non-deformable particles [47].

### In Vitro PDT of SMONs-HA-Ce6 and MONs-HA-Ce6

**Fig. 6:**
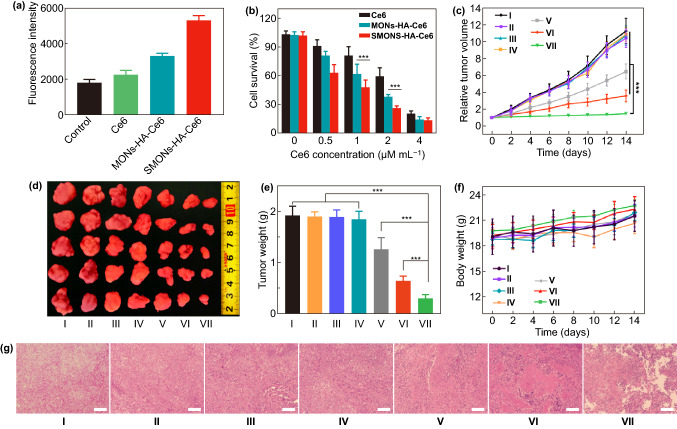
PDT efficacy. **a** Fluorescence intensity of DCF in MCF-7 cells, which incubated with different agents and then received laser irradiation (660 nm, 0.5 W cm^−2^, 5 min), measured by a microplate reader. **b** Viability of MCF-7 cells co-incubated with different concentrations of Ce6, MONs-HA-Ce6, SMONs-HA-Ce6 for 24 h and then irradiated with laser (660 nm, 0.5 W cm^−2^) for 5 min. **c** Tumor growth curves of different groups (*n* = 5 mice per group). **d** Tumor photographs and **e** tumor weights at the end point after various treatments. **f** The body weights of MCF-7 tumor-bearing mice during treatments. **g** H&E staining images of tissue sections from tumors of different groups at 24 h after irradiation. Scale bars, 100 µm. ***P < 0.001. I: PBS group; II MONs-HA-Ce6 group; III: SMONs-HA-Ce6 group; IV: PBS + laser group; V: free Ce6 + laser group; VI: MONs-HA-Ce6 + laser group; VII: SMONs-HA-Ce6 + laser group

Considering the advantageous biological behavior of the soft SMONs-HA-Cy5.5, we construct a photodynamic nanoplatform by loading a photosensitizer Ce6 in the SMONs-HA-Cy5.5. The loading capacities of the MONs-HA-Cy5.5 and SMONs-HA-Cy5.5 for Ce6 are measured to be 25.7 wt% and 29.9 wt%, respectively. The hemolytic activities of Ce6 loaded nanoplatforms are lower than 1% at a concentration of up to 400 μg mL^−1^, indicating excellent hemocompatibility (Fig. S12). The CCK8 cytotoxicity results indicate that the relative viability of MCF-7 cells remains over 80% after incubation with Ce6 loaded nanoplatforms (Fig. S13a). The ^1^O_2_ levels in the presence of these nanoplatforms were firstly detected in vitro. After irradiated by a 660 nm laser for 5 min, the SOSG fluorescence intensities of free Ce6, MONs-HA-Ce6 and SMONs-HA-Ce6 group are measured to be 41,626, 34,815, 35,918, respectively (Fig. S13b), suggesting the generation of ^1^O_2_. Also, the fluorescence intensity elevates gradually along with irradiation time (Fig. S13c, d). Then the production of ROS was studied at cellular level using DCFH-DA. The fluorescence intensity measured by a microplate reader was the highest when cells were incubated with SMONs-HA-Ce6, which demonstrates the ability of SMONs-HA-Ce6 to generate more ROS in cells (Fig. 6a). Next, a CCK-8 assay was carried out to evaluate their photodynamic effects on MCF-7 cells. After incubation with these nanoplatforms for 24 h and subsequently received irradiation of a 660 nm laser for 5 min, the relative cell viabilities show a dose-dependent decrease (Fig. 6b). More notably, the relative cell viabilities of SMONs-HA-Ce6 group are significantly lower than those treated with MONs-HA-Ce6 or free Ce6.

### In Vivo Antitumor Efficacy

Then, the in vivo PDT efficacy of the SMONs-HA-Ce6 was evaluated in MCF-7 tumor-bearing mice. As is illustrated in Fig. 6c, the tumor volumes of free Ce6 + laser group, MONs-HA-Ce6 + laser group and SMONs-HA-Ce6 + laser group are smaller compared to those of PBS, PBS + laser, MONs-HA-Ce6 and SMONs-HA-Ce6 groups, suggesting that PDT can inhibit tumor growth. In detail, the relative tumor volume of SMONs-HA-Ce6 + laser group is 1.46, while the PBS + laser group, free Ce6 + laser group and MONs-HA-Ce6 + laser group are 10.38, 6.46, and 3.57, respectively. It is obvious that SMONs-HA-Ce6 + laser group reaches an efficacy better than that offered by MONs-HA-Ce6 + laser while free Ce6 +laser group has the minimal tumor-suppressive efficacy. The significantly improved PDT efficacy of the SMONs-HA-Ce6 is attributed to their higher tumor accumulation and deeper penetration. Mice were killed on day 14, and the excised tumors were weighed. The average tumor weights of the PBS group, MONs-HA-Ce6 group, SMONs-HA-Ce6 group, PBS + laser group, free Ce6 + laser group, MONs-HA-Ce6 + laser group and SMONs-HA-Ce6 + laser group are 1.92, 1.90, 1.89, 1.84, 1.26, 0.64, and 0.30 g, respectively (Fig. 6d, e), further confirming that the SMONs-HA-Ce6 group is the most effective in inhibiting tumor growth. The body weights of mice from each group show no significant change, suggesting no major toxicity (Fig. 6f). The antitumor activities were further assessed by analyzing necrosis condition of H&E staining images of tumor sections at 24 h post-irradiation. Notably, H&E images show that enhanced necrosis occurs in tumor tissues of the mice treated with SMONs-HA-Ce6 + laser group compared with MONs-HA-Ce6 + laser group and free Ce6 + laser group, suggesting the improved efficacy of the soft photodynamic nanoplatforms in destroying tumor tissues (Fig. 6g). These results indicate that the soft mesoporous nanoplatform can effectively improve the tumor-suppressive efficiency.

## Conclusion

In summary, we successfully constructed soft mesoporous organosilica nanomaterials harboring a large cavity, cross-wrinkled morphology, and a low Young’s modulus (24.2 MPa). Analysis of their cellular internalization, blood circulation, tumor accumulation/penetration performances reveal that apart from a significantly improved efficiency of cellular uptake in MCF-7 cells, these nanomaterials also exhibit elongated blood circulation, with a fivefold increase in blood drug concentrations compared to stiff ones in the distribution phase, and exhibit drastically higher tumor accumulation. Strikingly, the soft SMONs-HA-Cy5.5 nanomaterials enable higher tumor penetration, reaching 30 μm-deeper tissue in 3D tumor spheroids. Furthermore, ex vivo intratumoral distribution results indicate that the soft mesoporous nanomaterials significantly improve extravasation behavior and intratumoral penetration, generating a 16-fold increase in diffusion distance in the tumor microenvironment (43 vs. 2.72 μm), relative to stiff counterparts.

Functionally, the soft deformable nanoparticles may have unique fluid mechanics that enable then to reside longer in the circulation system. A relatively longer blood circulation time enhances exposure of soft nanoplatforms to the tumor microvasculature allowing them to accumulate in the tumor site via the EPR effect. During extravasation of nanoparticles, from the systemic circulation to perivascular tumor microenvironment, it is easier for the soft ones to pass through leaky tumor vessels, owing to their deformed nature and improved tumor exposure. During penetration of the tumor parenchyma, soft nanoparticles could deform into ellipsoids, which may contribute to a rotational motion in complex crowded media. This explains why the soft nanoparticles diffuse faster and penetrate deeper into the dense tumor ECM compared to stiff counterparts.

Interestingly, after loading a therapeutic agent Ce6, the soft mesoporous organosilica nanoplatforms exhibit significantly improved MCF-7 cells killing effect in vitro and bring better therapeutic outcomes for breast cancer in vivo thanks to their higher tumor accumulation and deeper tumor penetration compared to the stiff ones. This is the first study comprehensively exploring the biological performance of soft mesoporous nanoplatforms. Overall, our findings demonstrate tremendous advantages of these nanomaterials, over their stiff counterparts, including improved blood circulation, tumor accumulation/penetration, and photodynamic efficacy. Consequently, the soft mesoporous nanoplatform, reported herein, has the potential for further future development of therapeutic anticancer approaches.

## Electronic supplementary material

Below is the link to the electronic supplementary material.

Supplementary material 1 (PDF 1001 kb)
