# Supplementary material for: Soft Mesoporous Organosilica Nanoplatforms Improve Blood Circulation, Tumor Accumulation/Penetration, and Photodynamic Efficacy
Source: Nanomicro Lett. 2020 Jun 30;12:137. doi: 10.1007/s40820-020-00465-7 (PMC7770801; doi:10.1007/s40820-020-00465-7)
Supplement: Supplementary file 1 — Supplementary material 1 (PDF 1001 kb) [file 40820_2020_465_MOESM1_ESM.pdf]

Supporting Information for

Soft Mesoporous Organosilica Nanoplatforms Improve Blood Circulation, Tumor Accumulation/Penetration, and Photodynamic Efficacy

Xin Peng[1, #], Kun Chen[3, #], Wanhua Liu[1, *], Xiongfeng Cao[4], Mengru Wang[1], Jun Tao[3], Ying Tian[2], Lei Bao[5], Guangming Lu[2, *], Zhaogang Teng[2, 3, *]

[1]Jiangsu Key Laboratory of Molecular and Functional Imaging, Department of Radiology, Zhongda Hospital, School of Medicine, Southeast University, Nanjing, 210009 Jiangsu, People's Republic of China

[2]Department of Medical Imaging, Jinling Hospital, School of Medicine, Nanjing University, Nanjing 210002, People's Republic of China

[3]Key Laboratory for Organic Electronics and Information Displays, Jiangsu Key Laboratory for Biosensors, Institute of Advanced Materials, Jiangsu National Synergetic Innovation Centre for Advanced Materials, Nanjing University of Posts and Telecommunications, Nanjing 210023, People's Republic of China.

[4]Affiliated Hospital of Jiangsu University, Jiangsu University, Zhenjiang 212001, People's Republic of China

[5]Soft Matter & Interface Group, School of Engineering, RMIT University, Melbourne, VIC 3000, Australia

#Xin Peng and Kun Chen contributed equally to this work

*Corresponding authors. E-mail: liuwanhua.com@126.com (Wanhua Liu); cjr.luguangming@vip.163.com (Guangming Lu); iamzgteng@njupt.edu.cn (Zhaogang Teng)

Supplementary Table and Figures

Table S1 Hydrodynamic diameter and zeta potential of the SMONs-HA-Cy5.5 and MONs-HA-Cy5.5 after storing in different solvent conditions for two weeks

	Ethanol		Water		PBS		DMEM(10%FBS)	
	Diameter	PDI	Diameter	PDI	Diameter	PDI	Diameter	PDI
SMONs-HA-Cy5.5	235.7±2.4	0.153	285.3±1.2	0.106	249.2±2.1	0.203	248.2±3.5	0.187
MONs-HA-Cy5.5	215.2±2.3	0.128	263.3±1.5	0.132	237.8±1.7	0.198	225.8±0.6	0.089

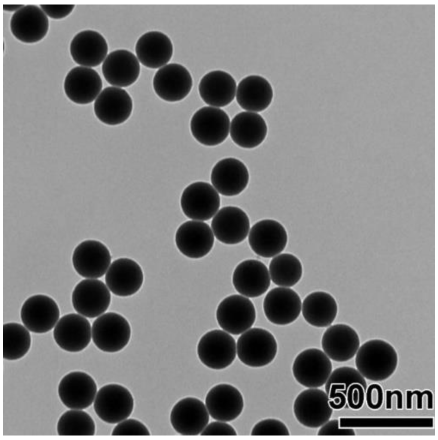

Fig. S1 TEM of the mother MONs prepared by *via* a CTAB-directed sol–gel process

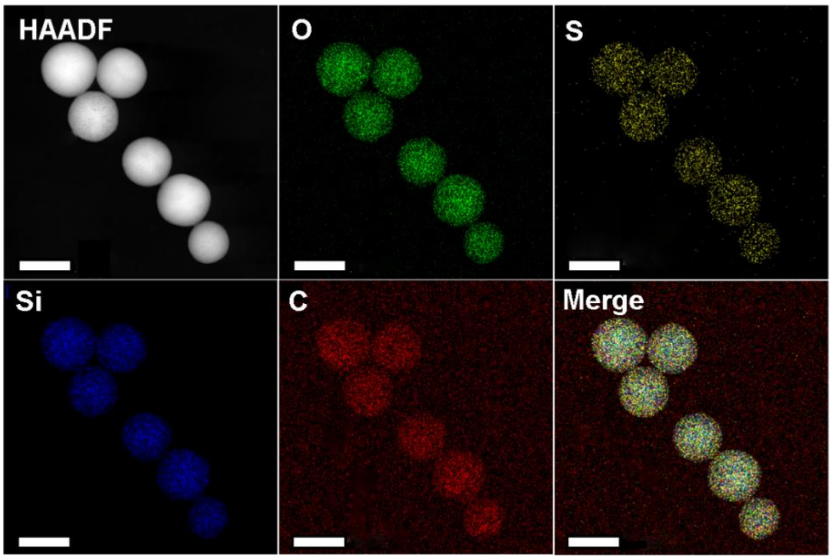

Fig. S2 STEM-HAADF image and EDX elemental mapping images of the MONs-HA-Cy5,5. Scale bars, 100 nm

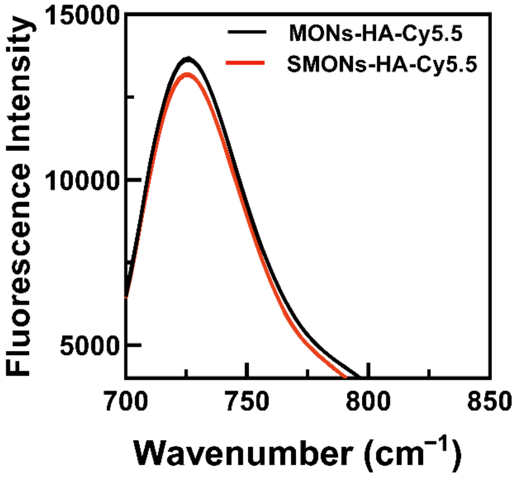

Fig. S3 Uv-vis of the SMONs-HA-Cy5.5 and MONs-HA-Cy5.5

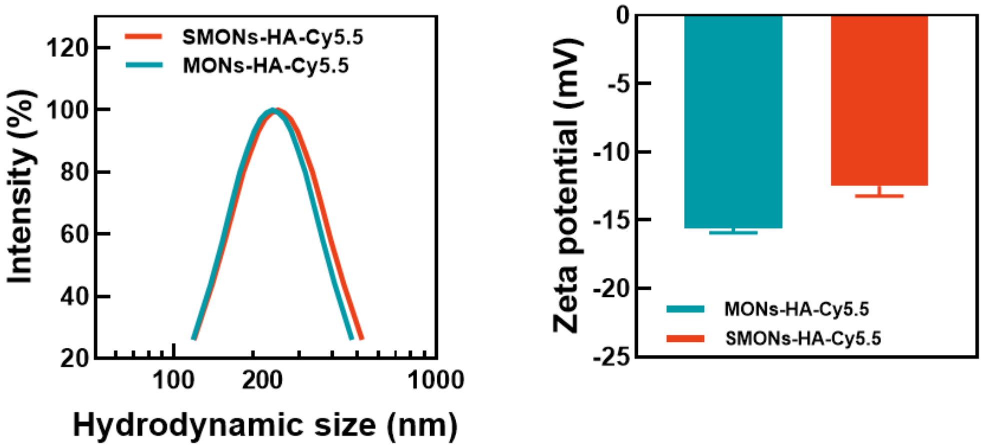

Fig. S4 The hydrodynamic diameters and zeta potential of MONs-HA-Cy5.5 and SMONs-HA-Cy5.5 after been stored in PBS for two weeks

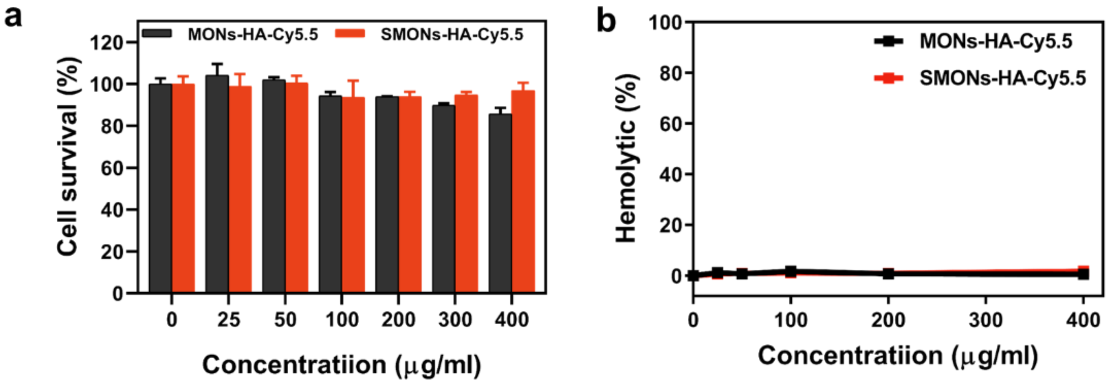

Fig. S5 (**a**) Cytotoxicity and (**b**) hemocompatibility of the MONs-HA-Cy5.5 and SMONs-HA-Cy5.5

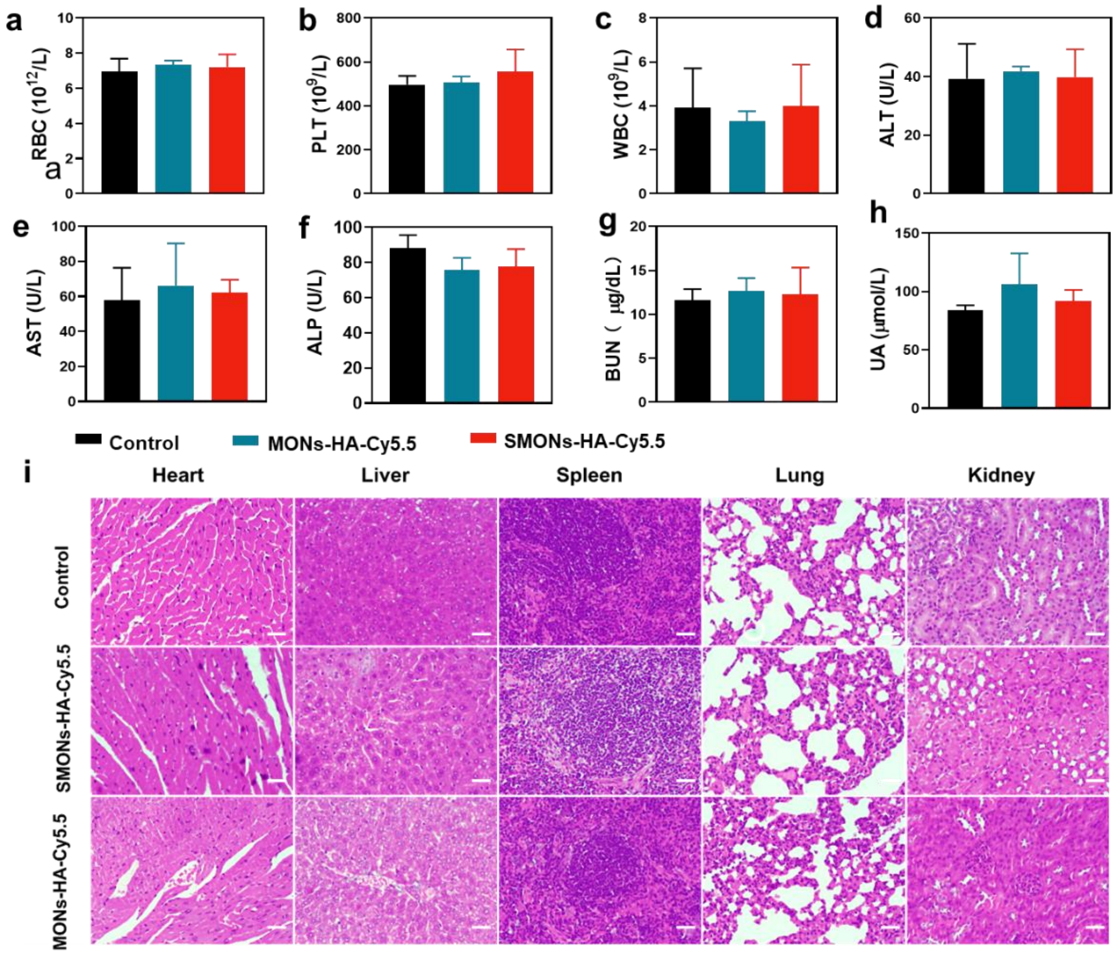

Fig. S6 Biocompatibility of the MONs-HA-Cy5.5 and SMONs-HA-Cy5.5 (**a**–**h**) Blood routine and serum biochemical analysis. (**i**) H&E staining of the major organs of mice (n = 3) intravenously injected with normal saline, MONs-HA-Cy5.5 and SMONs-HA-Cy5.5. Scale bars, 100 μm

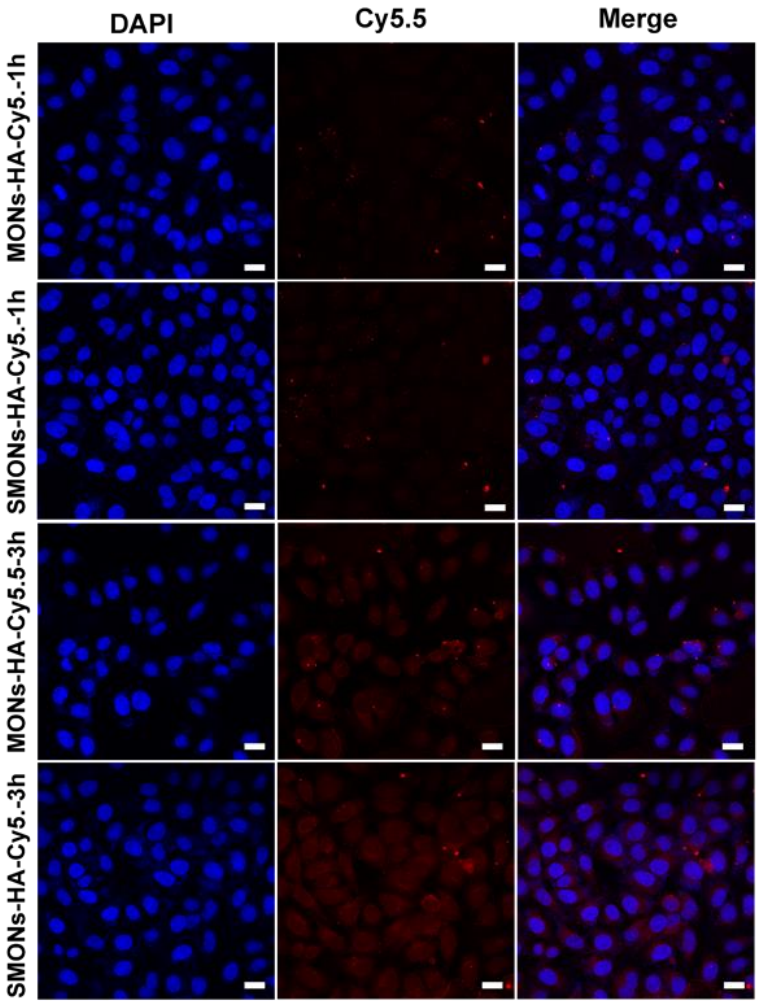

Fig. S7 CLSM images of MCF-7 cells incubated with MONs-HA-Cy5.5 and SMONs-HA-Cy5.5 for 1 and 3 h. Scale bars, 25 μm

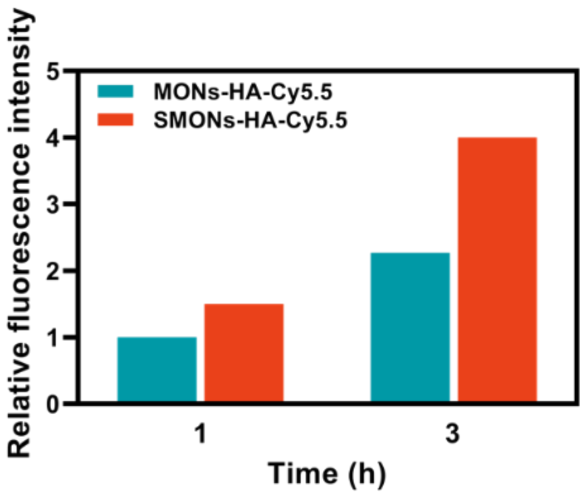

Fig. S8 Relative fluorescence intensity analysis of MCF-7 cells incubated with the MONs-HA-Cy5.5 and SMONs-HA-Cy5.5 for 1 and 3 h

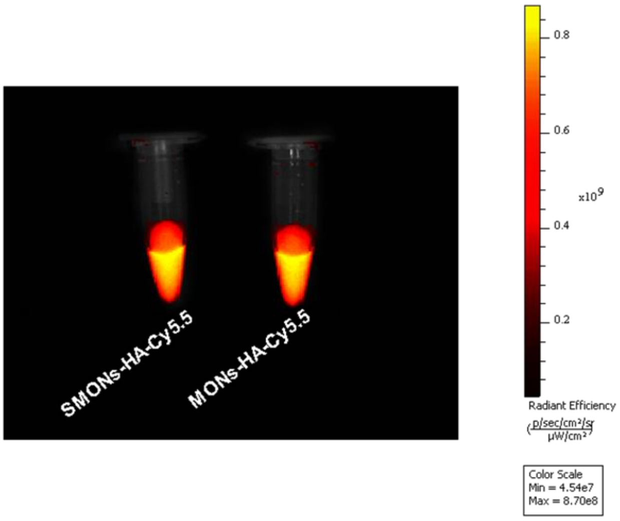

	Total Radiant Efficiency	Avg Radiant Efficiency	Stdev Radiant Efficiency	Min Radiant Efficiency	Max Radiant Efficiency
MONs-HA-Cy5.5	8.44×10^9	6.68×10^8	1.10×10^8	4.78×10^8	9.08×10^8
SMONs-HA-Cy5.5	8.54×10^9	6.64×10^8	1.12×10^8	4.58×10^8	8.77×10^8

Fig. S9 NIFR image and corresponding and quantification of the fluorescence intensity of PBS solutions containing SMONs-HA-Cy5.5 or MONs-HA-Cy5.5

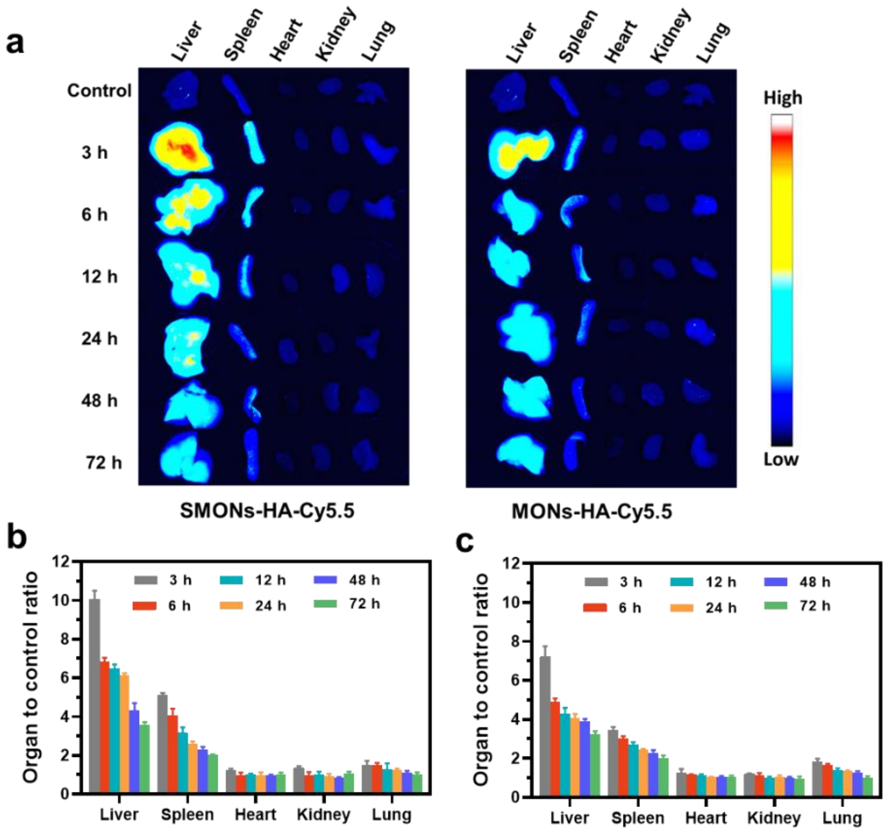

Fig. S10 a Ex vivo NIFR imaging and **b** quantification of the fluorescence intensity in each organ at different times after the administration of the SMONs-HA-Cy5.5 or MONs-HA-Cy5.5

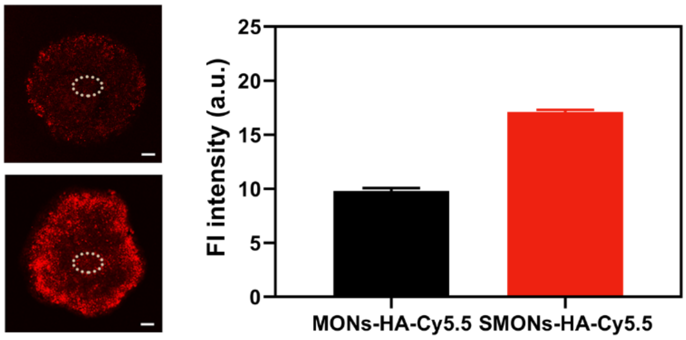

Fig. S11 Fluorescence intensity of the MCSs central region at the Z-axis distance of 30 μm

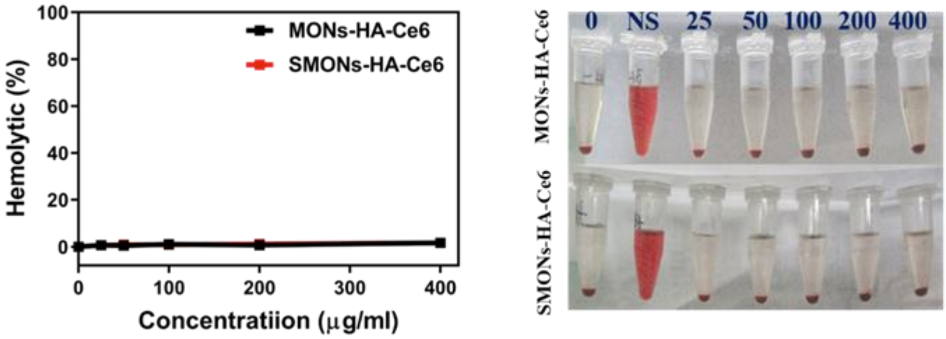

Fig. S12 Fluorescence intensity of the MCSs central region at the Z-axis distance of 30 μm

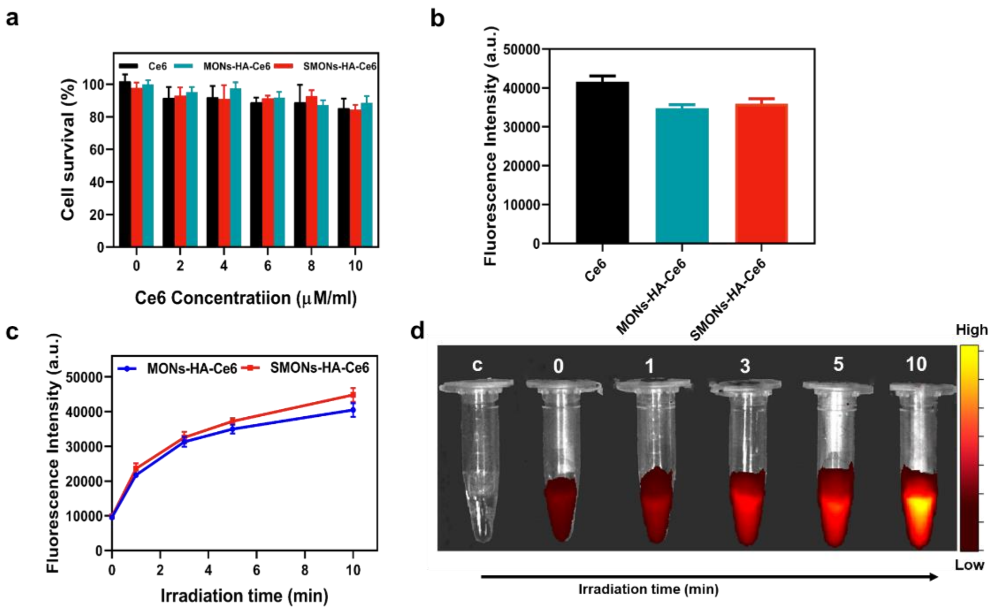

Fig. S13 (**a**) Cytotoxicity of Ce6, MONs-HA-Ce6 and SMONs-HA-Ce6. (**b-d**) Detecting the generation of ROS. (**b**) $^1O_2$ production of MONs-HA-Ce6 and SMONs-HA-Ce6 (4×10^{-6} M Ce6 equiv.) H_2O_2 (3 wt %) after irradiated (660 nm, 0.5 W cm^{-2}) for 5 min. (**c**) SOSG fluorescence signal of MONs-HA-Ce6 and SMONs-HA-Ce6 under different time periods after irradiation. (**d**) Corresponding near-infrared fluorescence imaging of SMONs-HA-Ce6 after irradiation for different time
